# Gestational Diabetes Risk and Low Birth Weight After Metabolic Bariatric Surgery: a Complex Interplay to be Balanced

**DOI:** 10.1007/s11695-024-07314-1

**Published:** 2024-06-04

**Authors:** Diana Rodrigues-Martins, Sara Andrade, Sofia S. Pereira, Jorge Braga, Inês Nunes, Mariana P. Monteiro

**Affiliations:** 1 Centro Materno-Infantil do Norte – Centro Hospitalar Universitário de Santo António (CMIN-CHUdSA), Porto, Portugal; 2 Endocrine and Metabolic Research, UMIB - Unit for Multidisciplinary Research in Biomedicine, ICBAS - School of Medicine and Biomedical Sciences, University of Porto, Porto, Portugal; 3 ITR - Laboratory for Integrative and Translational Research in Population Health, Porto, Portugal; 4 Obstetrics and Gynecology, Centro Hospitalar Vila Nova de Gaia/ Espinho, Porto, Portugal; 5 CINTESIS - Centro de Investigação Em Tecnologias E Serviços de Saúde, University of Porto, Porto, Portugal; 6 Institute of Biomedical Sciences Abel Salazar - University of Porto, Rua Jorge Viterbo Ferreira 228, Building 1.3, 4050-313 Porto, Portugal

**Keywords:** Pregnancy, Bariatric surgery, Perinatal outcomes

## Abstract

**Introduction:**

Metabolic bariatric surgery (MBS) is known to improve the obstetric outcomes of women with obesity and to prevent gestational diabetes (GD). To what extent does MBS decreases GD, without incurring at additional risks is a matter of concern.

**Methods:**

A retrospective case–control study to compare the pregnancy outcomes of women previously submitted to MBS to those of age and preconception body mass index (PC BMI) matched non-operated controls.

**Results:**

Pregnancies of women after MBS (*n* = 79) and matched controls (*n* = 79) were included. GD was significantly less frequent after MBS (7.6% vs. 19%; *p* = 0.03). Fasting blood glucose (76.90 ± 0.77 vs 80.37 ± 1.15 mg/dl, *p* < 0.05; 70.08 ± 1.34 vs. 76.35 ± 0.95 mg/dl; *p* < 0.05, first and second trimesters respectively) and birth weight (2953.67 ± 489.51 g vs. 3229.11 ± 476.21 g; *p* < 0.01) were significantly lower after MBS when compared to controls. The occurrence of small-for-gestational-age (SGA) was more frequent after MBS (22.8% vs. 6.3%; *p* < 0.01), but no longer significant after controlling for smoking habits (15.5% vs. 6%, *p* = 0.14). There were no significant differences in gestational weight gain, prematurity rate nor mode of delivery between groups.

**Conclusion:**

MBS was associated with a lower prevalence of GD than observed in non-operated women with the same age and BMI. After controlling for smoking, this occurred at the expense of a lower birth weight. Our data reinforces the hypothesis that MBS has body weight independent effects on glucose kinetics during pregnancy with distinctive impacts for mother and offspring, which need to be balanced.

**Graphical Abstract:**

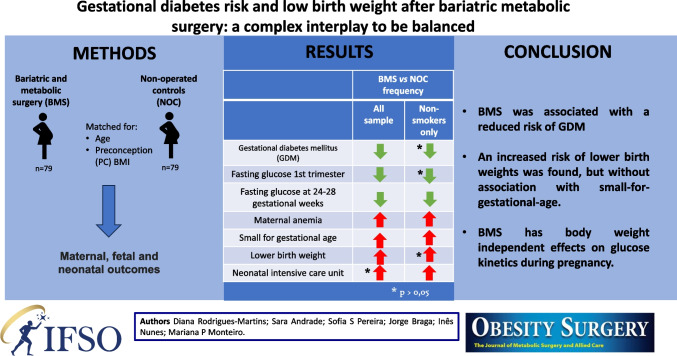

**Supplementary Information:**

The online version contains supplementary material available at 10.1007/s11695-024-07314-1.

## Introduction

In parallel to the rising prevalence of obesity in the general population, the prevalence of obesity in women during pregnancy is also increasing, which is estimated to range between 7 and 25% in Europe [1, 2]. Women with obesity are at increased risk of short- and long-term pregnancy complications for both mother and kindred [3]. Adverse obesity-related pregnancy outcomes include gestational diabetes (GD), hypertensive disorders of pregnancy (HDP), prematurity, cesarean delivery, perinatal mortality, and congenital malformations [4].

Given that metabolic bariatric surgery (MBS) is being increasingly performed in women at reproductive age, pregnancies after MBS are also becoming progressively more frequent [5, 6]. The available evidence shows that MBS is associated with a significant reduction in the risk of several obesity-associated diseases and most particularly GD [7]. However, concerns have also been raised on the potential harms, such as fetal growth restriction (FGR) and small-for-gestational-age (SGA) infants [8, 9]. In a recent meta-analysis, pregnant women after MBS matched for preconception (PC) BMI to non-operated controls were shown to be at increased risk of SGA infants and preterm delivery, suggesting that despite the benefits, surgery also carries additional risks. However, given that the risk for adverse obstetric outcomes was higher than observed in women matched for PC BMI, suggests that these could be independent of BMI [8]. Notwithstanding, data interpretation is hampered by the heterogeneity of populations, as most of the original evidence available on MBS and pregnancy outcomes derives from case–control studies [10] and the fact that controls were not always comparable regarding body mass index (BMI) and maternal age [11]. Consequently, it is not entirely clear whether the benefits, as well as the risks on maternal and neonatal outcomes, are solely the end result of weight loss or whether there is also a contributing role of surgery, itself. Therefore, aim was to compare the obstetric and neonatal outcomes of women previously submitted to MBS to those of non-operated women matched for age and PC BMI.

## Materials and Methods

### Study Design and Population

This was a retrospective case–control study that included pregnant women submitted to MBS and non-operated controls, admitted for prenatal care and delivery at a single public academic tertiary center, from January 2018 to December 2021. Twin pregnancies, pregnancies resulting from medical-assisted reproduction, or pregnancies which deliveries occurred at another institution, were pre-established exclusion criteria.

Electronic medical records were used for data retrieval. SGA was defined as birth weight < 10th percentile, based on race-ethnicity-, and sex-specific birth weights for gestational age cutoffs [12]. In non-operated controls, GD diagnosis was performed according to International Association of the Diabetes and Pregnancy Study Group guidelines and criteria, by assessing fasting glucose during the first trimester, which if normal (< 92 mg/dl) was followed by a subsequent screening test between 24–28 gestation weeks, with a 75 g oral glucose tolerance test (OGTT) [13]. In those women after MBS, not previously diagnosed with GD with fasting glucose at first trimester, capillary glucose self-monitoring before and after main meals up to six times daily for 2 weeks, between 24 and 28 gestational weeks was used given the OGTT limitation in this population.

### Control Matching Process

Potential non-operated controls were identified among pregnant women attending the center during the same time interval and selected for inclusion by age and PC BMI matching, implementing a propensity score analysis using the XLSTAT add-on for Microsoft Excel®. Thus, for each post-surgery delivery, one control delivery was matched by maternal PC BMI and maternal age at conception, in order to ensure homogeneity of the comparing groups.

### Outcomes

The pre-established primary outcome of this study was the occurrence of major obstetric complications, namely GD, HDP, SGA, and neonatal intensive care unit (NICU) admission. The secondary outcomes were gestational weight gain (GWG), mode of delivery, newborn body weight, and maternal complications during puerperium.

### Statistical Analysis

The statistical analysis involved descriptive statistics, namely absolute and relative frequencies, means and standard deviations, and inferential statistics. The normality of distribution was analyzed with the Shapiro–Wilk test and the homogeneity of variances with Levene’s test. The chi-square and Fisher’s tests were used to compare categorical variables. For the continuous variables, the Student’s *t*-test and Mann–Whitney test were used, depending on variables normality. According to variable category, linear or logistic regression analysis was performed to evaluate if pre-operative BMI was associated with pregnancy outcomes. A logistic regression analysis was used to identify predictors of SGA.

The significance level for rejecting the null hypothesis was set at (α) ≤ 0.05. Statistical analysis was performed with SPSS Statistics® program, version 28.0.

## Results

Women submitted to MBS prior to conception were identified among those admitted for antenatal care during the pre-specified time period (*n* = 91). After applying the exclusion criteria, 12 pregnancies were excluded for the following reasons: delivery at another institution (*n* = 9), pregnancy after assisted reproduction techniques (*n* = 2) and twin pregnancy (*n* = 1), yielding the final number of eligible pregnancies for analysis (*n* = 79), which were pair matched to controls (*n* = 79) as above described. Women who underwent MBS clinical features before surgery and conception are displayed at Table 1. Besides age and PC BMI, operated and non-operated women were also matched for other non-specified clinical features and despite smoking before and/or during pregnancy being more frequent in the MBS group, the difference was not statistically significant (26.6% *vs.* 15.2%; *p* = 0.08) (Table 2).

**Table 1:** Clinical features of women who underwent MBS prior to pregnancy

	Women submitted to MBS (*n* = 79)
Age at MBS (years)	27.52 ± 4.69
Body weight before MBS (kg)^A^	121.82 ± 16.28
BMI before MBS (kg/m^2^)^A^	45.20 ± 5.90
Obesity-associated diseases before MBS	
Type 2 diabetes	8 (10.1%)
Hypertension	15 (19%)
Dyslipidemia	8 (10.1%)
Type of bariatric procedure	
Roux-en-Y gastric bypass	44 (55.7%)
Sleeve gastrectomy	20 (25.3%)
Adjustable gastric banding	14 (17.7%)
Biliopancreatic diversion	1 (1.3%)
Total weight loss (%)^A^	33.70 ± 11.71
Total excess BMI loss (%)^A^	66.3 ± 11.71
Time between MBS and conception (months)	53.53 ± 47.84
Time between MBS and conception < 12 months	13 (16.5%)
Vitamin supplementation prior to conception	38 (48.1%)

^A^Missing data – *n* = 3; BMI – body mass index; MBS – metabolic bariatric surgery

**Table 2:** Clinical features of women who underwent MBS prior to conception compared to age and BMI matched controls

	Women after MBS (*n* = 79)	Control group (*n* = 79)	*p*
Age at conception (years)	31.87 ± 4.64	31.99 ± 4.68	0.88
Body weight before pregnancy (kg)	80.25 ± 15.12	79.71 ± 18.93	0.92
BMI before pregnancy (kg/m^2^)	29.76 ± 5.34	29.89 ± 6.91	0.91
Smoking habits (*)	21 (26.6%)	12 (15.2%)	0.08
Primiparous	39 (49.4%)	31 (39.2%)	0.2
Type 2 diabetes at conception	0**	0	1.0
Chronic hypertension at conception	4 (5.1%)	3 (3.8%)	1.0

BMI – body mass index; MBS – metabolic bariatric surgery. (*) Defined as smoking before conception regardless being active or inactive during pregnancy. **All women with diagnosed type 2 diabetes before MBS fulfilled the criteria for type 2 diabetes remission at the time of conception

Significant differences in the pre-specified pregnancy outcomes were identified between groups—namely GD was less frequent in the MBS group (7.6% *vs.* 19%; *p* = 0.03). First and second trimester fasting glucose, birth weight and newborn height were significantly lower in MBS group, while the frequency of SGA (22.8% *vs.* 6.3%; *p* < 0.01) was higher in MBS group compared to controls (Table 3). NICU admission occurred in four newborns of the MBS group (severe fetal growth restriction (*n* = 1), renal malformation with pulmonary dysplasia (*n* = 1), neonatal bradycardia (*n* = 1) and respiratory distress (*n* = 1)) and in a newborn of the control group (due to prematurity) (5.1% *vs.* 1.3%; *p* = 0.37). No neonatal deaths occurred. Maternal complications during puerperium were more frequent in the MBS group (17.7% *vs.* 5.1%; *p* < 0.01), being anemia and wound infections the most accountable entities (*p* = 0.01 and *p* = 0.03 respectively) (Table 3). There were no significant differences between groups regarding gestational age at delivery, induction of labor or mode of delivery.

**Table 3:** Obstetric and neonatal outcomes of women after MBS compared to matched controls

	Pregnancies after MBS (*n* = 79)	Pregnancies in non-operated controls (*n* = 79)	*p*
GWG (kg)	12.19 ± 7.41	11.60 ± 6.61	0.6
Fasting plasma glucose at first trimester (mg/dl)^a^	76.90 ± 0.77	80.37 ± 1.15	**0.03**
Fasting plasma glucose at 24–28 gestational weeks (mg/dl)^b^	70.08 ± 1.34	76.35 ± 0.95	** < 0.01**
GD	6 (7.6%)	15 (19%)	**0.03**
HDP	7 (8.9%)	8 (10.1%)	0.79
SGA	18 (22.8%)	5 (6.3%)	** < 0.01**
Anemia	29 (36.7%)	11 (13.9%)	** < 0.01**
Intra-venous iron treatment	22 (27.8%)	1 (1.3%)	** < 0.01**
ASA during pregnancy	52 (65.8%)	31 (39.2%)	** < 0.01**
Pre-term delivery	6 (7.6%)	4 (5.1%)	0.51
Gestational age at delivery (weeks)	38.95 ± 1.63	39.20 ± 1.48	0.28
Induced labour	24 (31.2%)	19 (24.1%)	0.32
Cesarean delivery	28 (35.4%)	28 (35.4%)	1.0
Emergent cesarean section^c^	14 (51.9%)	20 (74.1%)	0.09
Birth weight (g)	2953.67 ± 489.51	3229.11 ± 476.21	** < 0.01**
Birth weight > 4000 (g)	2 (2.5%)	2 (2.5%)	1.0
New-born´s height (cm)	47.93 ± 2.31	48.83 ± 2.74	**0.01**
APGAR < 7 at 5th minute	0	1 (1.3%)	1.0
Neonatal death	0	0	-
NICU	4 (5.1%)	1 (1.3%)	0.37
Puerperium complications Anemia Wound infection Hypertension	14 (17.7%) 9 (11.4%) 4 (5.1%) 1	4 (5.1%) 2 (2.5%) 2 (2.5%) 0	** < 0.01** **0.01** **0.03** 0.45
Intravenous iron or RBC transfusion during puerperium	5 (6.3%)	1 (1.3%)	0.21

^a^16 missing/not eligible data on both pregnancy after BMS and control groups; ^b^32 missing/not eligible data on pregnancy after BMS group and 12 on control group; ^c^ n = 56 (28 on pregnancy after BMS group and 28 on control group)

ASA—Acetylsalicylic acid; BMI – Body Mass Index; MBS – Metabolic Bariatric Surgery; GD – Gestational Diabetes; SGA – Small-for-Gestational-Age; HDP – Hypertensive Diseases of Pregnancy; NICU – Neonatal Intensive Care Unit; IV – Intravenous; RBC – Red Blood Cells

On regression analysis, pre-operative BMI was not found to be significantly associated with any of the pregnancy outcomes (supplementary Table 1), while smoking was the only variable found to be associated with a higher risk of SGA (supplementary Table 2). Hence, a subanalysis excluding pregnancies from smoking mothers was then performed to disentangle the influence of this variable on pregnancy outcomes. From this subanalysis, fasting glucose at 24–28 gestational weeks, birth weight and birth height remained significantly lower; however, the SGA frequency was no longer statistically different in the MBS group when compared to control group (15.5% *vs.* 6%; *p* = 0.14) (Table 4). A sub-group analysis comparing pregnancies of women who conceived < 12 months vs > 12 month after MBS to evaluate the potential impact on birth weight and the occurrence of SGA was performed, but no significant differences were found between the groups (supplementary Table 3).

**Table 4:** Obstetric and neonatal outcomes of pregnancies of non-smoking women after BMS compared to matched controls

	Pregnancies after MBS (*n* = 58)	Pregnancies in non-operated controls (*n* = 67)	*p*
Fasting glucose at first trimester (mg/dL)^a^	77.14 ± 0.93	80.31 ± 1.29	0.1
Fasting glucose at 24–28 gestational weeks (mg/dL)^b^	69.57 ± 1.51	76.19 ± 1.07	** < 0.01**
SGA	9 (15.5%)	4 (6%)	0.14
Birth weight (g)	2996 ± 67	3264 ± 56	** < 0.01**
New-born´s height (cm)	48.1 ± 0.3	49 ± 0.3	**0.03**
NICU	4 (6.9%)	0	**0.04**

^a^11 missing/not eligible data on pregnancy after BMS group and 12 on control groups; ^b^18 missing/not eligible data on pregnancy after BMS group and 10 on control group

BMI – Body Mass Index; MBS – Metabolic Bariatric Surgery; SGA – Small-for-Gestational-Age; NICU – Neonatal Intensive Care Unit

## Discussion

MBS is a highly effective weight loss intervention and weight loss decreases the risk of adverse obstetric outcomes in women with obesity. However, to what extent is MBS able to revert the risk of obesity-associated obstetric complications towards the levels of age and BMI matched women, without incurring at additional surgical-related risks, is not entirely known [3, 7]. Therefore, to address this knowledge gap, we decided to compare the pregnancy outcomes of women previously submitted to MBS to those of non-operated women matched for age and PC BMI.

Our study shows that GD was significantly less frequent, while birth weight was significantly lower after MBS when compared to age and PC BMI matched controls, after controlling for smoking.

Our case–control study matched for PC BMI, which contrasts to previous studies that used pre-surgery BMI as control [14], allowed us to further explore and disentangle the effects of MBS at reducing the incidence of GD beyond the weight loss achieved. At first glance, our study findings corroborate the previously available evidence on GD after MBS [7, 15, 16]. Furthermore, despite there were no differences regarding PC BMI and age, our finding of a lower fasting glucose, which exerts a major influence over GD risk, supports the hypothesis that during pregnancy MBS also influences glycemic dynamics in a body weight independent manner.

Nevertheless, the so far available evidence on GD and MBS must be interpreted with caution, as the majority of the studies used a 75 g or 100 g OGTT for GD diagnosis [17], despite the known caveat of depicting altered glucose kinetics, with early hyperglycemia leading to an increased rate of false positive GD diagnoses [18]. Indeed, fasting glucose levels were previously shown to be lower in women after MBS when compared both to lean or BMI-matched controls, while the postprandial glucose excursion curve showed a characteristic pattern, including a glucose rise at 60 min followed by reactive hypoglycemia, which occurred in 54.8% of the cases [19]. Although there is no doubt that alternative GD diagnostic criteria are still needed for this specific population [18], in the meantime, frequent capillary blood glucose self-monitoring is the diagnostic resource most frequently used [20]. The fact that in our study GD screening relied on capillary blood glucose measurements further strengthens the finding of a reduced prevalence of GD after MBS, irrespectively of the weight loss achieved or GWG.

MBS was also associated with a higher prevalence of SGA and reduced newborn’s birth weight and height, despite there were no significant differences on GWG or time from surgery to conception. Despite the difficulties to differentiate a SGA from FGR fetus [21], the association between MBS and low birth weight, regardless of the matching performed, seems to be fairly unanimous [9, 22–24]. Kwong et al. found that the risk of SGA in women after BMS was higher when compared to women matched for pre-surgery BMI, as well as, PC BMI with an odds ratio (OR) of 2.16 and an OR of 2.23, respectively [8]. In a subsequent study by Jacamon et al., MBS was associated with a reduced risk of excessive fetal growth with a trend for a higher incidence of SGA, despite matching on PC BMI [15]. Another study concluded that in singleton gestations, women with obesity who underwent a prior MBS, had a higher risk of FGR, which further supports the association of MBS with FGR, regardless the weight loss or BMI achieved [25]. The lack of essential nutrients in result of a combination of food restriction and malabsorption was hypothesized to be responsible for the negative impact on fetal growth [26]. A short surgery-to-conception interval and a GWG lower than recommended by the Institute of Medicine (IOM) are additional factors that may influence fetal growth after MBS [14, 23]. Moreover, a greater glycemic variability, a lower glucose nadir during the OGTT and a greater incidence of postprandial hypoglycemia were also proposed to have a negative impact on the development of fetuses from women previously submitted to gastric bypass [27]. Nevertheless, detailed data on glucose kinetics during pregnancies after different BMS procedures are not currently available [19]. Continuous glucose monitoring during pregnancy after BMS could not only prove to be a valid alternative to diagnose GD, but also provide detailed information on glucose kinetics, most particularly the occurrence of asymptomatic hypoglycemic events, and how these impact on pregnancy outcomes [28]. Moreover, this data could be useful to guide clinicians and dieticians to provide targeted macronutrient intake counseling to avoid hypoglycemic events, potentially harmful to the fetus [29].

When managing pregnancy in women after MBS it is important to take into account several factors, obesity related or not, which may influence the obstetric outcomes [30, 31]. Smoking is recognized to be a major risk factor for lower fetal weight, irrespective of mother’s surgical status [32]. After excluding smoking mothers from the pregnancies analysis, there was no longer a significant difference in SGA, although both fasting glucose mean and birth weight remained significantly lower.

Daily low-dose ASA is part of standard obstetric care to reduce the risk for preeclampsia, preterm birth, small for gestational age/intrauterine growth restriction, and perinatal mortality in pregnant women at high risk for preeclampsia [33]. Pregnant women submitted to MBS frequently fulfil criteria for ASA prescription (previous history of preeclampsia, BMI greater than 30, maternal age greater or equal to 35 years) [34]. As a consequence, the use of ASA was more prevalent in women submitted to surgery.

Maternal anemia during gestation and puerperium was also more frequent in pregnancies of women who had undergone MBS. In our study, less than half of the women previously submitted to MBS reported to adhere to vitamin supplement recommendations prior to conception, and more than one in ten women conceived within one year after the surgery despite medical counselling to postpone pregnancy plans until after the second postoperative year. Although these women were under regular follow-up with a multidisciplinary obesity team, it has been clearly shown that most patients either fail to perceive the potential benefits of vitamin supplementation compliance or have financial constraints, which render micronutrient deficiencies a frequent complication after MBS [11],

The main strengths of this study are the adjustment for two major variables for the outcomes being studied (age and PC BMI), the fact that PC BMI and not pre-surgery BMI was considered, and that all women were followed up at a single center by the same multidisciplinary team comprising obstetricians, endocrinologists and dietitians. The GD screening was performed by frequent capillary glucose self-monitoring; data on fasting glucose was included in the analysis, and the impact of smoking was considered on obstetrics outcomes. However, there are also limitations that must be acknowledged. First, the study retrospective design and small sample size are likely to have hindered the statistical significance of some parameters. Second, not all women underwent MBS at the same institution nor by the same surgical team. Third, given the small numbers, it was not possible to evaluate a potential influence of different types of MBS procedures on the study outcomes. Forth, it was not possible to evaluate to what extent the outcomes found were due to the PC BMI rather than residual from the pre-surgery BMI. Finally, although smoking arises as an important variable accounting for the occurrence of SGA, we were unable to disentangle the relative contribution of active vs inactive smoking due to lack of data.

## Conclusions

MBS was associated with a prevalence of GD that was lower than observed in non-operated women with the same age and BMI. After controlling for smoking habits, this occurred at the expense of a lower birth weight but without criteria for SGA. Our data reinforces the hypothesis that MBS has body weight independent effects on glucose kinetics during pregnancy with potentially distinctive impacts for mother and offspring, which need to be balanced.

### Supplementary Information

Below is the link to the electronic supplementary material.

Supplementary file1 (DOCX 16 KB)

Supplementary file2 (DOCX 16 KB)

Supplementary file3 (DOCX 18 KB)
