# Supplementary material for: Gestational Diabetes Risk and Low Birth Weight After Metabolic Bariatric Surgery: a Complex Interplay to be Balanced
Source: Obes Surg. 2024 Jun 4;34(7):2546–52. doi: 10.1007/s11695-024-07314-1 (PMC11217113; doi:10.1007/s11695-024-07314-1)
Supplement: Supplementary file 1 — Supplementary file1 (DOCX 16 KB) [file 11695_2024_7314_MOESM1_ESM.docx]

**Supplementary Table 1 – Univariate regression analysis regarding the impact of the pre-operatory body mass index in the pregnancy outcomes.**

|  | **Univariate analysis** | |
| --- | --- | --- |
|  | **OR (95% CI)** | **p value** |
| GD | -2.311 (-7.321; 2.699) | 0.361 |
| HDP | 3.987 (-2.027; 10.000) | 0.191 |
| SGA | 0.647 (-2.546; 3.839) | 0.688 |
| Anemia | 0.100 (-2.716; 2.917) | 0.944 |
| Pre-term delivery | 1.446 (-3.581; 6.473) | 0.568 |
| Gestational age at delivery (weeks) | -0.289 (-1.128; 0.550) | 0.494 |
| Induced labour | 0.330 (-2.679; 3.339) | 0.828 |
| Cesarean delivery | -0.357 (-1.318; 0.603) | 0.461 |
| Emergent cesarean section^c^ | 0.960 (-4.651; 6.572) | 0.726 |
| Birth weight (gr) | -0.002 (-0.004; 0.001) | 0.238 |
| NICU | 9.304 (3.615; 14.994) | **0.002** |

GD – Gestational Diabetes; HDP – Hypertensive Diseases of Pregnancy; SGA – Small-for-Gestational-Age; NICU – Neonatal Intensive Care Unit.
