# Supplementary material for: Gestational Diabetes Risk and Low Birth Weight After Metabolic Bariatric Surgery: a Complex Interplay to be Balanced
Source: Obes Surg. 2024 Jun 4;34(7):2546–52. doi: 10.1007/s11695-024-07314-1 (PMC11217113; doi:10.1007/s11695-024-07314-1)
Supplement: Supplementary file 2 — Supplementary file2 (DOCX 16 KB) [file 11695_2024_7314_MOESM2_ESM.docx]

**Supplementary Table 2 – Univariate and multivariate regression analysis regarding the influence of the patients’ characteristics before conception and the Small-for-Gestational-Age.**

|  | **Univariate analysis** | | **Multivariate analysis** | |
| --- | --- | --- | --- | --- |
|  | **OR (95% CI)** | **p value** | **OR (95% CI)** | **p value** |
| Age at conception (years) | 0.254 (0.898; 1.126) | 0.924 |  |  |
| Body weight before pregnancy (kg) | 0.993 (0.967; 1.020) | 0.597 |  |  |
| BMI before pregnancy (kg/m^2^) | 0.983 (0.914; 1.058) | 0.651 |  |  |
| Smoking habits | 3.746 (1.465; 9.576) | **0.006** | 3.894 (1.486; 10.207) | **0.006** |
| Primiparous | 2.727 (1.082; 6.872) | **0.033** | 2.843 (1.097; 7.369) | **0.032** |
| Type 2 diabetes at conception | 4.071 (0.905; 18.323) | 0.067 |  |  |
| Chronic hypertension at conception | 4.071 (0.905; 18.323) | 0.067 |  |  |

BMI – Body Mass Index
