# Supplementary material for: Gestational Diabetes Risk and Low Birth Weight After Metabolic Bariatric Surgery: a Complex Interplay to be Balanced
Source: Obes Surg. 2024 Jun 4;34(7):2546–52. doi: 10.1007/s11695-024-07314-1 (PMC11217113; doi:10.1007/s11695-024-07314-1)
Supplement: Supplementary file 3 — Supplementary file3 (DOCX 18 KB) [file 11695_2024_7314_MOESM3_ESM.docx]

**Supplementary Table 3 – Sub-group analysis of women with conception <12 months vs >12 months after MBS**

|  | <12months after BMS (n=13) | >12months after BMS (n=66) | P |
| --- | --- | --- | --- |
| Birth weight (gr) | 2948 ± 65 | 2981 ± 58 | 0.625 |
| Newborn´s height (cm) | 47.8 ± 0.3 | 48.4 ± 0.3 | 0.396 |
| SGA | 2 (15.4%) | 16 (24.2%) | 0.775 |

SGA- Small for gestational age
